# Deciphering taxon-specific metabolic shifts during pathogenic fungal infection in leaf-cutter ant fungal gardens

**DOI:** 10.1093/ismeco/ycag213

**Published:** 2026-07-23

**Authors:** Marija Veličković, Margaret W Thairu, Yuqian Gao, Chaevien S Clendinen, Nathalie Munoz, Priscila M Lalli, Carrie D Nicora, Kelly G Stratton, Matthew E Monroe, Ronald J Moore, Cameron R Currie, Ruonan Wu, Kristin E Burnum-Johnson

**Affiliations:** The Environmental Molecular Sciences Laboratory, Pacific Northwest National Laboratory, Richland, WA 99354, United States; Wisconsin Institute for Discovery and Department of Plant Pathology, University of Wisconsin-Madison, Madison, WI 53715, United States; Biological Sciences Division, Pacific Northwest National Laboratory, Richland, WA 99354, United States; The Environmental Molecular Sciences Laboratory, Pacific Northwest National Laboratory, Richland, WA 99354, United States; The Environmental Molecular Sciences Laboratory, Pacific Northwest National Laboratory, Richland, WA 99354, United States; Biological Sciences Division, Pacific Northwest National Laboratory, Richland, WA 99354, United States; Biological Sciences Division, Pacific Northwest National Laboratory, Richland, WA 99354, United States; The Environmental Molecular Sciences Laboratory, Pacific Northwest National Laboratory, Richland, WA 99354, United States; Biological Sciences Division, Pacific Northwest National Laboratory, Richland, WA 99354, United States; Biological Sciences Division, Pacific Northwest National Laboratory, Richland, WA 99354, United States; Department of Bacteriology, University of Wisconsin-Madison, Madison, WI 53706, United States; Department of Biochemistry & Biomedical Sciences, McMaster University, Hamilton, ON, L8S 4L8, Canada; Biological Sciences Division, Pacific Northwest National Laboratory, Richland, WA 99354, United States; The Environmental Molecular Sciences Laboratory, Pacific Northwest National Laboratory, Richland, WA 99354, United States

**Keywords:** integrative multi-omics, metaproteomics, metabolomics, leaf-cutter ant fungal garden, perturbation

## Abstract

Leaf-cutter ant fungal gardens are a living library of bioeconomy solutions, where the mutualist fungus *Leucoagaricus gongylophorus* drives lignocellulose degradation and conversion into valuable bioproducts. However, these gardens are vulnerable to infection by the specialized pathogen *Escovopsis weberi*, which disrupts garden stability. Despite their ecological importance, the metabolic mechanisms underlying fungal–fungal competition and community-level responses to infection remain poorly understood. To address this gap, we experimentally infected six fungal garden consortia with *E. weberi*, establishing an infection gradient within each consortium, which we then systematically analyzed using a multi-omics approach. Metabolomic profiling revealed infection-induced alterations in metabolite abundance, while metaproteomic analysis identified fungal-specific shifts in protein levels across a spatiotemporal scale. The integration of multi-omics datasets across all fungal garden consortia provided a comprehensive view of the underlying pathways, revealing that the pathogenic fungus exploits nutrient supplies derived from lignocellulose degradation by native fungi. Reconstruction of active pathways enabled us to trace nutrient flow and map taxon-specific metabolic shifts in response to infection. Specifically, a distinct switch from lysine synthesis by native fungi to lysine catabolism orchestrated by pathogenic fungi. These findings offer a system-level perspective of the molecular alterations driving competitive interactions between native and pathogenic species within the fungal garden consortium. This study not only highlights the power of multi-omics approaches in unraveling the complexity of microbial consortia across ecological scales but also provides an integrated road map to efficiently harness microbiome data for deeper insights into microbial interactions and community responses to perturbations.

Leaf-cutter ants obtain nutrients by cultivating a symbiotic fungus *Leucoagaricus gongylophorus* on freshly harvested leaves [1]. This fungus, along with other microbial partners, forms a fungal garden consortium that apart from decomposing plant matter to release nutrients for sustaining ants, also produces an array of secondary metabolites, many of which have bioactive properties to maintain ecological balance [2–6]. The efficiency of the consortium can be disrupted by the necrotrophic mycoparasite *Escovopsis*, which invades the fungus garden and devastates it by eventually overrunning the native fungal cultivar, ultimately resulting in colony collapse [7]. Understanding how infections perturb these complex microbial communities requires mechanistic insights into the molecular and metabolic dynamics of community members. To address this, we collected reference samples from healthy fungal gardens following the experimental infection of six independent consortia. Each garden was placed in a tube and the pathogenic fungus *Escovopsis weberi* was introduced on one side, enabling the pathogen to gradually proliferate toward the center (Fig. 1a). This experimental design created an infection gradient with contrasting microbial compositions on the two sides of the consortium. To unravel the taxon-specific molecular alterations and associated metabolic shift driving these dynamic responses across spatiotemporal scales in the context of early infection, we subdivided each consortium into smaller infection-related groups (ranging from 9 to 11 groups, depending on the consortium). We conducted advanced metaproteomic and metabolomic analyses as detailed in the Materials and Methods section and Supplementary Figs S1–S4 in Supplementary Information.

**Figure 1:**
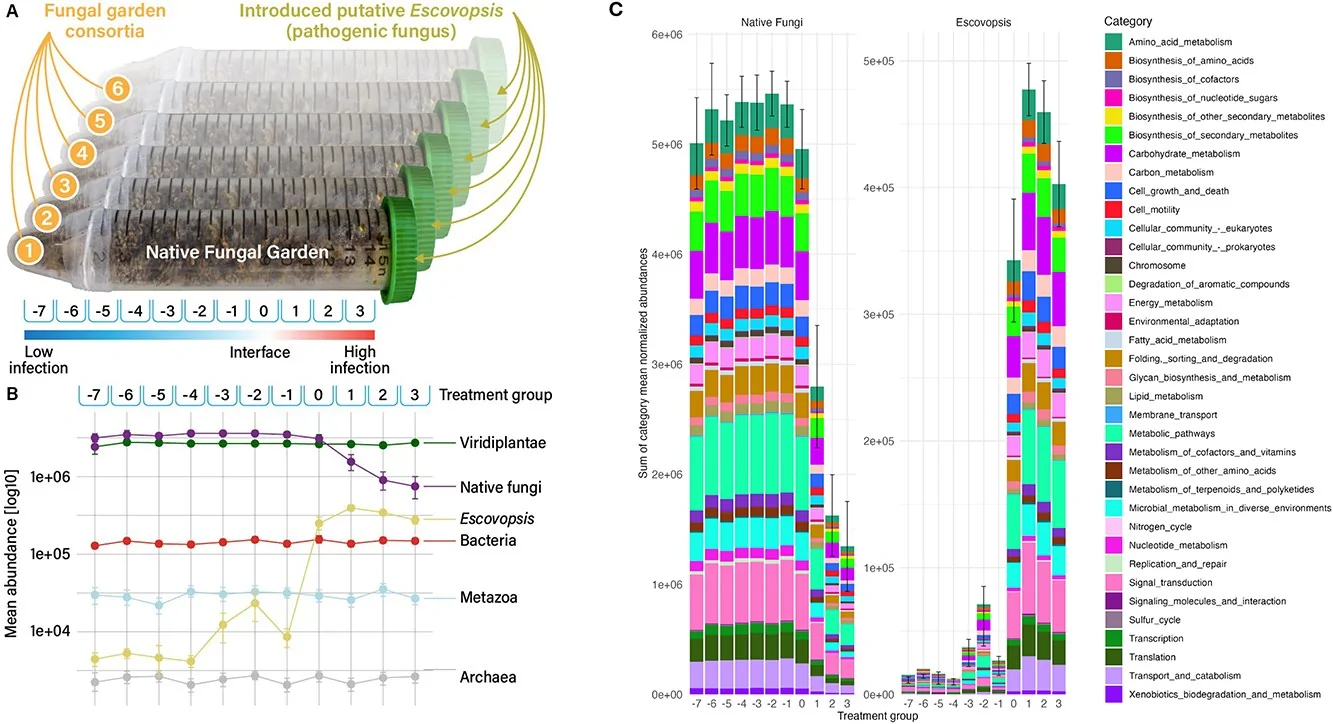
Spatiotemporal taxonomic and functional characterizations of heterogeneity in naturally evolved fungal garden consortia under infection-induced perturbation conditions. (a) An example image of six native fungal garden consortia infected by the pathogenic fungus *Escovopsis weberi* on one side of the system. The image illustrates a spatiotemporal gradient of infection across fungal garden consortium tubes, realigned so their interfaces (treatment Group 0) overlapped (Supplementary Fig. S3). This delineates the low-infection zone (treatment Groups −7 to −1) and the high-infection zone (treatment Groups 1–3). (b) Metaproteomics-informed taxa distribution across the tubes, highlighting dynamic changes in taxa abundance based on peptide profiles in response to infection-induced perturbation. The line plot represents the mean abundance of detected taxon-specific peptides, with error bars indicating the standard deviation of treatment group replicates from all six fungal garden consortia. (c) Metaproteomics-informed functional annotation showing the distribution of pathway categories along the infection gradient, associated with native and pathogenic fungal species, based on data from all six fungal garden consortia. Each colored segment represents the mean normalized abundance of a pathway category across replicate samples within a treatment group and taxon, and total bar height represents the sum of these category means. Error bars indicate variation among replicate samples in the sample-level total normalized abundance across categories, shown as standard error.

Fungal garden consortia represent complex microbial ecosystems characterized by wide taxonomic diversity and dynamic interactions among community members [5]. Through comprehensive metaproteomic profiling, this study uncovers infection-driven shifts in molecular dynamics of community members, shedding light on the spatiotemporal changes triggered by the mycoparasite *Escovopsis* and its competitive interplay with native fungi. As such, our metaproteomics profiling detected 61 473 unique peptide sequences attributed to 300 732 proteins, which were subsequently mapped to 69 271 protein clusters across all treatment group samples from six fungal garden consortia. Taxonomic annotations of these clusters revealed complex and diverse population profiles in consortia, with highly abundant plant material dominated by native fungi. Coexisting taxa (*Escovopsis*, Bacteria, Metazoa, and Archaea) were present with lower abundances. Spatial profiling uncovered distinct patterns of protein and peptide abundances among microbial consortia members along the infection gradient. As depicted in Fig. 1b and Supplementary Fig. S4, the patterns observed across all six fungal garden infection experiments were consistent. The introduction of *Escovopsis* triggered a dynamic spatiotemporal shift, characterized by its increased proliferation at the side of introduction, and coinciding with a gradual decline in the growth of the native fungi. This dynamic delineated three distinct zones: the native garden experiencing low infection (Groups −7 to −1), the interface (Group 0), and the high-infection (Groups 1–3). In contrast, other microbial taxa remained stable, with no significant changes in peptide or protein abundance across the infection zones. This indicates that fungal–fungal interactions drive the molecular dynamics of early infection, while broader microbial community responses emerge over longer timescales. This aligns with previous work showing that *Burkholderia*, isolated from a fungus garden, inhibits the parasitic fungus *Escovopsis* on timescales longer than those captured in our study [3]. The coexisting taxa also exhibited high peptide-level redundancy with other community members, whereas the fungal taxa showed distinct and well-resolved peptide signatures, with 1739 protein clusters confidently assigned to *Escovopsis* and 6701 to the native fungi, compared with far fewer for non-fungal groups. Because our study focuses on the microbial members whose molecular responses are supported by robust, taxon-specific protein identifications, we centered our biological interpretation on the fungal members, as they provided the clearest and the highest-confidence metaproteomic resolution at the early infection stage. Accordingly, herein we highlight the molecular interplay between native fungus and garden mycoparasite, *Escovopsis*, enabling deeper insights into the molecular dynamics underlying competition and coexistence between these two fungal species.

Functional characterization of metaproteomic data provided a comprehensive overview of metabolic processes across a spatiotemporal scale, revealing a high overlap between native and pathogenic fungi pathway categories (Fig. 1c). Both fungal species showed a high abundance of proteins associated with amino acid processing, biosynthesis of secondary metabolites, and pathways powered by lignocellulose degradation (e.g., carbohydrate metabolism, transport and catabolism, signal transduction). Although they showed high pathway category overlap, abundant carbohydrate-active enzymes specifically for lignin, hemicellulose, and pectin polymer decomposition were detected mainly in native fungi (Supplementary Fig. S5). This confirms that native fungi are the primary drivers of plant cell wall degradation. These observations align with previously published findings, which demonstrated that *Escovopsis* generally exhibits reduced genome size and gene content specialized for host attack, and the exploitation of host-derived nutrients [8, 9]. Interestingly, the abundance of most lignocellulose-degrading enzymes produced by native fungi remained relatively stable along the infection gradient, likely due to the infection being in its early stage. These stable dynamics of lignocellulose decomposition imply continuous availability of low-molecular-weight lignocellulose-related products, indirectly supporting the survival and proliferation of *Escovopsis*. To further investigate these dynamics, we integrated metaproteomic and metabolomics data to elucidate the downstream metabolic processing of lignin-related aromatic compounds, tracing nutrient flow in response to perturbations.

Our multi-omics integration results strongly indicate that *Escovopsis* actively transforms lignin-related aromatic compounds into catechol in the interface and high-infection zones, generating energy and precursor molecules essential for biosynthesis. As depicted in Fig. 2a, phenol and 2,3-dihydroxybenzoate are converted to catechol by phenol 2-monooxygenase (K03380, EC 1.14.13.7) and 2,3-dihydroxybenzoate decarboxylase (K14333, EC 4.1.1.46), respectively. Subsequently, *Escovopsis* metabolizes catechol through the β-ketoadipate pathway, catalyzed by catechol 1,2-dioxygenase (K03381, EC 1.13.11.1). These findings align with previous demonstrations that genes encoding catechol dioxygenases are more abundant in some pathogenic fungi with a necrotrophic lifestyle than in saprophytic species [10]. Additionally, perturbation in the native fungal community led to diminished metabolomic processing of phenolic compounds (i.e. salicylate) to catechol via salicylate hydroxylase (K00480, EC: 1.14.13.1), resulting in the accumulation of salicylate in the infected zone.

**Figure 2:**
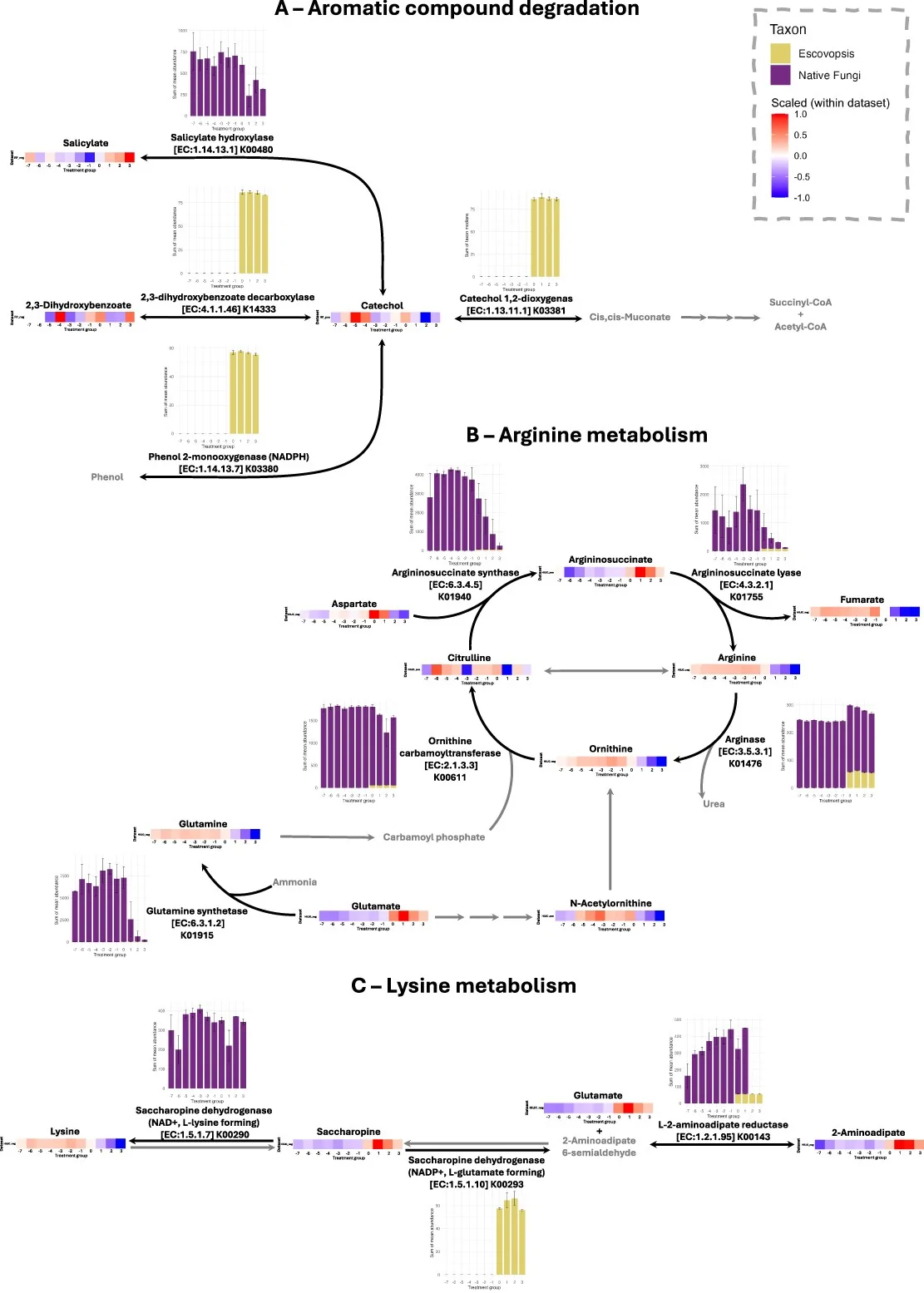
Multi-omics integration across all six fungal garden consortia unravels taxon-specific metabolic shifts within complex microbiomes over spatiotemporal scales. (a) Reconstructed pathways for aromatic compound degradation. (b) Reconstructed arginine metabolism pathway. (c) Reconstructed lysine metabolism pathway. The bar graph illustrates abundance change of the specific enzyme assigned to the two contrasting taxa, native fungus and *Escovopsis*, across the infection tube. Protein abundance is shown as the mean abundance across all six garden consortia for each treatment group. Error bars indicate variation among replicate samples for each taxon-specific segment, shown as standard error and positioned relative to the corresponding stacked segment. Metabolite heatmaps visualize dynamic changes in metabolite abundances along the infection gradient indicating the separation technique corresponding to the most confident identification for that compound. The heatmap summarizes metabolite abundance as the mean abundance across all six garden consortia for each treatment group. To ensure comparability across different separation techniques, these means were scaled within the compound-specific dataset, producing normalized values between −1 and 1. The infection gradient within the tube is divided into three zones: low-infection (−7 to −1), interface (0), and high-infection (1–3). Detailed pathway information, including peptide heatmaps visualizing the relative abundance of peptides associated with each discussed protein, metabolite heatmaps showing trends across all separation techniques in which the metabolites were detected, examples of individual garden consortium trends for metabolites, as well as levels of metabolite identification confidence, are provided in Supplementary Fig. S7.

Metaproteomic data showed highly abundant amino acid processing in both fungal species, while complementary metabolomics analyses highlighted significant changes in the abundance of specific amino acids (proline, lysine, arginine, phenylalanine, glutamine, tyrosine, and histidine) across the infection gradient (Supplementary Fig. S6). Elevated levels of amino acids in the low-infection zone suggest that the native fungus actively synthesizes amino acids not only to sustain its growth and biomass production but also to support metabolic functions crucial for the ant colony. The interface and high-infection zones showed notably reduced amino acid levels, suggesting that the competing pathogenic fungi alter amino acid metabolism by redirecting it toward catabolic processes or utilizing these amino acids for the synthesis of proteins and enzymes critical for survival. Through our integrative multi-omics approach, we investigated taxon-specific amino acid alteration on a spatiotemporal scale.

Knowing that ants have lost the ability to synthesize arginine and rely on their native fungal symbionts for its production [11], we specifically focused on reconstructing the arginine metabolomic pathway. As depicted in Fig. 2b, introduction of *Escovopsis* led to a notable reduction in the abundance of native fungal enzymes involved in arginine metabolism in both the interface and high-infection zones. This enzyme depletion corresponded with a reduction in arginine and corresponding metabolites within these regions. The presence of *Escovopsis* also disrupts nitrogen assimilation and the conversion of glutamate to glutamine. This alteration was evidenced by comparing the abundance of glutamine synthetase (K01915, EC: 6.3.1.2) and corresponding substrate and product across the different tube zones.

Additionally, we examined perturbations in lysine metabolism (Fig. 2c), given its important role in fungal growth and proliferation. In the low-infection zone, native fungi predominantly synthesize lysine through the α-aminoadipate pathway. This inference is supported by the accumulation of lysine and the detection of the enzyme saccharopine dehydrogenase (NAD+, l-lysine forming; K00290, EC:1.5.1.7), which catalyzes the conversion of saccharopine to lysine. Conversely, in the interface and high-infection zones, *Escovopsis* appears to reverse this pathway, as indicated by the accumulation of saccharopine and its subsequent degradation by a different form of saccharopine dehydrogenase (NADP+, l-glutamate forming; K00293, EC:1.5.1.10) enzyme. These results illuminate how infection-induced perturbation impacts taxon-specific shifts in amino acid metabolic processing.

The spatially resolved design was essential for uncovering where interactions between the two fungi unfold along the gradient by tracking how each fungus’s metabolic response changes across it, providing mechanistic insight into the underlying processes, such as a switch from amino acid biosynthesis by the native fungus in the low-infection zone to amino acid catabolism by *Escovopsis* in the high-infection zone. This spatial resolution enabled us to capture species-specific metabolomic patterns that would not be detectable in bulk or non-spatial designs. For our multi-omics integration, we focused on metabolites that were confidently integrated into pathways by pairing them with the corresponding enzymes that were uniquely assigned to a fungal taxon, with no redundancy from other microbial community members. While the majority of metabolites showed abundance patterns that followed the patterns of their associated enzymes, some metabolites showed less consistent trends, likely due to their involvement in interconnected metabolic networks. This variability was expected given the complexity of metabolic pathways and the shared intermediates across different biochemical processes. Collectively, these results demonstrate the power of our integrative multi-omics approach to resolve taxon-specific spatiotemporal metabolic shifts during perturbation, providing systems-level insights essential for engineering resilience and enhancing productivity in future bioeconomy applications.

## Supplementary Material

Web_Material_ycag213

## Data Availability

Raw proteomics and metabolomics data can be found via the Mass Spectrometry Interactive Virtual Environment (MassIVE; https://massive.ucsd.edu) under accession number MSV000099592. The analyzed metaproteome and metabolome datasets are available at Zenodo (https://zenodo.org/uploads/17049489; DOI: 10.5281/zenodo.17049489). Scripts are deposited on the PNNL GitLab (https://tanuki.pnnl.gov/ruonan.wu/kbj_doe_ecrp_bigfungalgarden).
