# Supplementary material for: Deciphering taxon-specific metabolic shifts during pathogenic fungal infection in leaf-cutter ant fungal gardens
Source: ISME Commun. 2026 Jul 23;6(1):ycag213. doi: 10.1093/ismeco/ycag213 (PMC13499455; doi:10.1093/ismeco/ycag213)
Supplement: Supplementary file 1 [file web_material_ycag213.docx]

**Supplementary Information**

**Deciphering taxon-specific metabolic shifts during pathogenic fungal infection in leaf-cutter ant fungal gardens**

Marija Veličković^1^, Margaret W Thairu^2^, Yuqian Gao^3^, Chaevien S. Clendinen^1^, Nathalie Munoz^1^, Priscila M Lalli^3^, Carrie D Nicora^3^, Kelly Stratton^1^, Matthew E. Monroe^3^, Ronald J Moore^3^, Cameron R Currie^4, 5^, Ruonan Wu^3, *^, and Kristin E Burnum-Johnson^1, *^

^1^The Environmental Molecular Sciences Laboratory, Pacific Northwest National Laboratory, Richland, WA, USA

^2^Wisconsin Institute for Discovery and Department of Plant Pathology, University of Wisconsin-Madison, Madison, WI, USA

^3^Biological Sciences Division, Pacific Northwest National Laboratory, Richland, WA, USA

^4^Department of Bacteriology, University of Wisconsin-Madison, Madison, WI, USA

^5^Department of Biochemistry & Biomedical Sciences, McMaster University, Hamilton, Ontario, Canada

Table of contents:

Materials & methods………………. page 2

Supplementary Figures…………… page 10

References………………………….… page 23

**Materials & methods**

**Laboratory-reared colonies and tube infection experiment**

Six fungal garden consortia samples were obtained from laboratory-maintained *Atta cephalotes* colonies: Garden 1 (April-A colony), Garden 2 (Bianca-A colony), Garden 3 (Joan-B colony), Garden 4 (Mona Lisa-A colony), Garden 5 (Mona Lisa-B colony), and Garden 6 (Donna-A colony). Therefore, these six garden consortia samples represent five independent colonies, each a biological replicate, and the Mona Lisa colony was sampled twice. Each colony was named after the queen, since queen-right leafcutter ant colonies in laboratory settings are maintained as monogynous (single-queen) systems. These colonies were reared on a mix of red maple (*Quercus rubra*) and red oak (*Acer rubrum*) leaves. For each garden, a reference sample was collected prior to transferring a middle stratum of the garden into 15 mL tubes. Gardens were inoculated by placing a 6-mm fungal plug of *Escovopsis weberi* at one end of each 15 mL tube. The progression of *E. weberi* infection was monitored twice daily until clear hyphal growth was observed in the center of the tube. This occurred after varying durations: 3 days for Gardens 4 and 6, 4 days for Gardens 1–3, and 5 days for Garden 5. **Supplementary Figure 1** provides visual documentation of each garden on the day the infection was ended. Once the infection period concluded, each infected garden was removed from the 15 mL tube and subdivided into ~1 cm sample fragments (i.e., treatment groups) along the infection gradient. This resulted in the formation of 9 samples for Garden 6, 10 samples for Gardens 1 and 3, and 11 samples for Gardens 2, 4, and 5.

**Metabolite and protein extraction**

Fungal garden samples were lyophilized and ground into a fine powder using a HG-600 Geno/Grinder® 2010 Tissue Homogenizer and Cell Lyser (Cole Palmer, Metuchen, NJ). Metabolites were extracted from 20 mg of ground tissue using the Metabolite, Protein, and Lipid extraction (MPLEx)[1-3] protocol. Briefly, powdered tissue was resuspended in Type 1 water and a cold (−20 °C) chloroform:methanol mixture (2:1, v/v) in chloroform-compatible 2 mL Sorenson MulTI™ SafeSeal™ microcentrifuge tubes (Sorenson Bioscience, Salt Lake City, UT). 2 mg from each sample was combined for use as a pool QC. Tubes were placed in an ice block, resulting in a final solvent ratio of 8 parts chloroform, 4 parts methanol, and 3 parts water per sample. Samples were vortexed, probe-sonicated on ice for 20 s at 20% amplitude (FB505, FisherBrand, Waltham, MA), and incubated in a Thermomixer (Eppendorf, Hamburg, Germany) at 1,000 rpm for 30 min. Phase separation was achieved by centrifugation at 12,000 × g for 10 min, yielding a polar phase, a non-polar phase, and a protein interlayer.

For metabolite recovery, the entire upper polar phase and one-quarter of the lower non-polar phase were collected into glass vials. The protein interlayer was re-extracted by adding 150 µL of water, followed by bath sonication on ice, addition of 750 µL chloroform:methanol (2:1, v/v), vortexing, and centrifugation as above. The polar phase and one-quarter of the non-polar phase were again pooled with the initial metabolite extract. The remaining protein interlayer was washed with cold 100% methanol, vortexed, and centrifuged for 5 min to pellet the protein. Protein pellets were lightly dried under a nitrogen stream and stored at −80 °C until digestion.

Metabolite samples were dried in a vacuum concentrator (Labconco, Kansas City, MO) and stored at −20 °C until analysis using liquid chromatography-tandem mass spectrometry (LC-MS/MS) and gas chromatography-mass spectrometry (GC-MS) analysis. For each metabolite sample (in glass MS vials) 600 µL of methanol:chloroform (4:1, v/v) was added. Samples were sonicated to resuspend metabolites, then shaken at 4 °C for 10 min at 750 rpm. From each sample, 480 µL of extract was transferred into new labeled MS vials, dried under vacuum, and stored at −20 °C for GC–MS analysis. The remaining 120 µL of extract was dried in the original sample vials, reconstituted in 130 µL of 80% methanol, and sonicated into solution for LC-MS/MS. Reconstituted samples were filtered through a conditioned 96-well 0.2 µm PTFE filter plate (AcroPrep™ Advance 96-well filter plates for solvent filtration, Cytiva, Marlborough, MA) using a positive pressure system (Waters, Milford, Massachusetts) into a 96-well collection plate. Filtrates were transferred into labeled Thermo SureSTART level 3 vials (Thermo Fisher, Waltham, Massachusetts), capped, and stored at −20 °C until analysis.

**Protein Digestion**

The protein interlayer was resuspended in 8 M urea prepared in 100 mM ammonium bicarbonate (pH 8). Protein concentration was determined using a bicinchoninic acid (BCA) assay (Thermo Scientific, Waltham, MA, USA). Following quantification, disulfide bonds were reduced with 10 mM dithiothreitol (DTT) at 60 °C for 30 min with continuous agitation at 800 rpm in a Thermomixer (Eppendorf, Hamburg, Germany). The urea concentration was then diluted to 4 M with 100 mM ammonium bicarbonate, and lysyl endopeptidase (Lys-C; Wako Fujifilm, Osaka, Japan) was added at a protein:Lys-C ratio of 10:1. After incubation at 37 °C for 4 h, the sample was further diluted to 1 M urea with 100 mM ammonium bicarbonate, and sequencing-grade trypsin (Promega, Madison, WI) was added at a protein:trypsin ratio of 50:1. Digestion was carried out overnight at 37 °C with shaking at 800 rpm, followed by automatic cooling to 4 °C after 16 h.

Following digestion, peptides were acidified to 0.5% trifluoracetic acid (TFA) and were desalted using a 4-probe positive pressure Gilson GX-274 ASPEC™ system (Gilson Inc., Middleton, WI) equipped with Discovery C18 100 mg/1 mL solid-phase extraction (SPE) columns (Supelco, St. Louis, MO). Columns were conditioned with 3 mL methanol, equilibrated with 2 mL of 0.1% trifluoroacetic acid (TFA) in H₂O, and loaded with the peptide samples. Bound peptides were washed with 4 mL of 95:5 H₂O:acetonitrile (ACN) containing 0.1% formic acid (FA) and eluted with 1 mL of 80:20 ACN:H₂O containing 0.1% FA.

Eluates were concentrated to ~100 µL using a vacuum concentrator (Labconco, Kansas City, MO), and peptide concentration was determined via BCA assay. The clean peptides were vialed at 0.1 µg/µL with nano pure water for LC-MS/MS peptide analysis.

**Peptide analysis using tandem mass spectrometry**

A Waters nano-Acquity dual pumping UPLC system (Milford, MA) was configured for on-line trapping of a 5 µL injection at 5 µL/min for 10 min followed by reverse-flow elution onto the analytical column at 200 nL/min. The trapping column was slurry packed in-house using 360 µm o.d. x 150 um id fused silica (Polymicro Technologies Inc., Phoenix, AZ) Jupiter 5µm C18 media (Phenomenex, Torrence, CA) with 2mm sol-gel frits on either end. The analytical column was slurry packed in-house using Waters BEH 1.7 µm particles packed into a 35cm long, 360 um od x 75 um id column with an integrated emitter (New Objective, Inc., Littleton, MA). Mobile phases consisted of (A) 0.1% formic acid in water and (B) 0.1% formic acid in acetonitrile with the following gradient profile (min, %B): 0, 1; 10, 8; 105, 25; 115, 35; 120, 75; 123, 95; 129, 95; 130, 50; 132, 95; 138, 95; 140,1.

MS analysis was performed using an Thermo Eclipse mass spectrometer (Thermo Scientific, San Jose, CA) equipped with a front-end High Field Asymmetric Waveform Ion Mobility Spectrometry (FAIMS) interface. The ion transfer tube temperature and spray voltage were 300ºC and 2.4 kV, respectively. Data was collected for 120 min following a 27 min delay from when the trapping column was switched inline with the analytical column. FAIMS was used at CVs -40V, -60V, and -80V. FT-MS spectra were acquired from 300-1800 m/z at a resolution of 120k (AGC target 4e5) and while the top 12 FT-HCD-MS/MS spectra were acquired in data dependent mode with an isolation window of 0.7 m/z and at an orbitrap resolution of 50K (AGC target 5e4) using a fixed collision energy (HCD) of 32 and a 30 sec exclusion time.

**Customized protein database for peptide tandem mass spectrometry data annotation**

The customized protein database, described previously[4] was constructed to maximize taxonomic and functional coverage of the leaf-cutter ant fungal garden microbiome. It integrates proteins translated from assemblies of leaf-cutter ant fungal garden metagenomes deposited at the JGI data portal and genomes of resident microbes[5-7], together with protein sequences predicted by de novo metaproteomics[8]. All sequences were dereplicated at 90% identity using CD-HIT (v4.8.1), yielding 24,809,256 non-redundant protein clusters. The longest sequence in each cluster was designated the representative cluster protein. This curated database served as the reference for annotating the high-resolution tandem mass spectrometry spectra with stringent matching criteria. To illustrate the comprehensiveness of our database with respect to taxonomic composition, we made a cross‑lineage phylogenetic tree (**Supplementary Figure 2).**

**Peptide tandem mass spectrometry data analysis**

MS/MS data were searched against our customized protein database with a decoy database of reversed sequences using MS-GF+ search engine (PMID: 25358478). Only peptides that were fully tryptic and had no more than two missed cleavages were retained for optimization. The spectrum-level peptide confidence score of the peptide-spectrum match (PSM), represented as MSGFDB_SpecProb in MS-GF+, and the precursor peptide ion mass error (in ppm), represented as DelM_PPM in MS-GF+, were optimized to maximize peptide identification in each dataset while maintaining a peptide-level target-decoy-based false discovery rate (FDR) below 1%. Peptides with only a single PSM and proteins with only one qualified peptide were also filtered out. After applying these stringent criteria, we confidently detected: 61,473 unique peptides, and 300,732 proteins, which were subsequently mapped to 69,271 protein clusters. Even with stringent filtering and clustering, some peptide redundancy is unavoidable in metaproteomics, and this can lead to inflated counts of protein clusters in certain cases. However, our peptide filtering, protein inference, and protein cluster assignment followed commonly accepted community standards for metaproteomic analyses[9, 10].

We implemented our published workflow with improvements to make functional and taxonomic assignments to the cluster proteins with high-confidence peptides detected. In brief, we integrated protein-level evidence with sequence similarity and contig-based context to maximize annotation accuracy while retaining conservative taxonomic assignments, and employed a profile Hidden Markov model (HMM) method for functional annotations. The cluster protein sequences were queried against the NCBI cluster_nr database using DIAMOND blastp (v2.16.0+; evalue cutoff of 10^-3^) and retained the top five hits for each query sequence. All the retained top hits with percent identity within 10 % points of this top hit were considered co-equal candidates. Taxonomic assignment was performed on the co-equal candidates using a lowest common ancestor (LCA) rule designed to balance specificity and conservatism. This rule reflects the uncertainty associated with highly similar homologs, ensuring that ambiguous cases are not over-assigned to an incorrect lineage. Cluster proteins lacking valid NCBI taxonomy identifiers were further examined by a contig-based method. We tracked back unannotated proteins to their originating contigs and then classified them using Contig Annotation Tool (CAT, v5.2.3) in contigs mode against the GTDB and NCBI reference databases. Functional annotation was performed by running KofamScan (v1.3.0) against the cluster protein sequences to assign KEGG Orthologs and pathways. This combined protein- and contig-based workflow maximized annotation coverage while retaining conservative taxonomic assignments through the LCA rule. The integration of protein abundance estimates at the cluster level ensured compatibility with quantitative metaproteomic analyses, while the functional annotation step allowed direct linkage of taxonomy and metabolic potential.

Functional annotation was used to examine changes in protein and peptide abundances of *Escovopsis* and native fungi along the infection gradient in each tube. For every tube, we identified the interface zone (treatment group 0) as the position where *Escovopsis* showed a clear transition in abundance across the tube and delineated the high- and low-infection zones (**Figure 1** and **Supplementary Figure 3**). After independently determining this interface with the proteomic data for each garden, we realigned all gardens so that their treatment group 0 positions overlapped (**Figure 1** and **Supplementary Figures 3-4**). Following alignment, 2 garden tubes spanned from −7 to +3 relative to the interface (yielding 11 standardized treatment groups), with the remaining garden tubes having slightly fewer groups. Because the infection in Garden 5 (Mona Lisa‑B) progressed the longest, its high‑infection zone extended further along the tube resulting in 6 treatment groups, hence samples M_B_9, M_B_10, and M_B_11 were removed to standardize the treatment groups from the lowest infection point (group -4 in this case) through the highest infection point (group 3).

We implemented a staged workflow to propagate peptide-level evidence through protein clusters to conservative taxonomic and functional labels, and to produce normalized quantitative estimates for downstream integration analyses. We first joined peptide intensities with peptide-protein relationships and the previously resolved protein-to-cluster mappings with taxonomic and functional annotations. To address baseline and putative contaminant signals, we excluded peptides observed in the healthy controls when their assignments indicated *Escovopsis*, thereby minimizing carry-through of likely non-target peptides into treatment contrasts. The peptide-level filtering and normalization were performed with pmartR (v2.0) to obtain intensity matrices and corresponding metadata. Peptide-level data were log_2_-transformed and subjected to stringent filtering, retaining only proteins supported by at least two peptides and peptides observed in at least two samples with sufficient non-missing values (≥2 per group for ANOVA, ≥3 for g-tests); outliers were further identified using robust Mahalanobis distance across correlation, missingness, dispersion, and distributional metrics, after which global median normalization was applied to minimize technical variation while preserving group structure. Protein aggregation (roll-up) was then executed in a custom Python script for computational efficiency, while preserving the pmartR statistical framework before and after aggregation. Protein-level normalization and statistics were computed by pmartR (v2.0). For integrative analyses, peptide- and protein-level normalized matrices were reshaped to long format, merged with sample grouping and the metadata tables, and exported alongside compact tables that retain only standardized identifiers, normalized abundances, and essential annotations. To focus on microbial processes and reduce off-target signal, we removed human-associated and other non-microbial pathways using a curated blocklist. The filter was applied uniformly to peptide and protein compact tables, producing pathway-cleaned outputs. All the input and key output tables with respective scripts are described in Data and Code availability and are publicly available on Zenodo and Gitlab.

Our metaproteome integrative analyses were designed to 1) quantify how taxonomic composition changes across treatments, 2) attribute functional signal to taxa and taxonomic signal to pathway categories, and 3) interrogate selected KOs for lineage-specific peptide evidence. All steps were executed in a custom R script using the normalized peptide and protein compact tables mentioned above. In brief, we quantified taxonomic dynamics across treatment groups by summing normalized peptide and protein abundances within replicates to the taxon level, then summarizing per treatment group as mean abundance ± standard error**.** Functional context was added by decomposing abundances by functional category composition within taxa and by taxonomic composition within functional categories in total normalized abundance per group. For KO-focused analysis, we combined protein-level KO abundance per taxon by summing normalized values with peptide-level evidence, where only the lineage-specific or taxon-unique peptides were included for visualization (**Figure 2** and **Supplementary Figures 5 and 7**). Together, these analyses provided robust taxonomic trends, conservative lineage-to-function relationships, and KO-specific taxon-unique peptide comparison.

**Gas chromatography–mass spectrometry**

Metabolites were derivatized by the method previously reported[4]: 20 μL of methoxyamine in pyridine (30 mg/mL) was added to each sample, followed by incubation at 37°C with shaking for 90 min to protect carbonyl groups. Next, 80 μL of N-methyl-N-(trimethylsilyl)trifluoroacetamide (MSTFA) with 1% trimethylchlorosilane (TMCS) was added to each vial, followed by incubation at 37°C with shaking for 30 min to derivatize hydroxyl and amine groups. The samples were then allowed to cool to room temperature and were analyzed in an Agilent GC 7890A coupled with MSD 5975C mass spectrometer (Agilent Technologies, Santa Clara, CA). Separations were performed on a HP-5MS column (30 m × 0.25 mm × 0.25 μm; Agilent Technologies). The injection mode was splitless, and the injection port temperature was held at 250 °C. The column oven was initially maintained at 60 °C for 1 min and then ramped to 325 °C by 10 °C/min, followed by a 10 min hold at 325 °C. The retention indices were first calculated based on the analysis of a mixture of fatty acid methyl esters (FAMEs), then this retention indexing information was subsequently applied to data from each sample to enable proper chromatographic alignment of each data set. Global metabolomic analysis was performed and data processing was carried out using MetaboliteDetector[5], including chromatographic alignment of all datasets, to search data from detected metabolites against entries in a PNNL augmented version of Agilent GC-MS metabolomics library, containing spectra and validated retention indices for more than 1200 metabolites. The NIST20/Wiley11 GC-MS library was used to cross-validate metabolite identifications made using the Agilent library, and to also identify metabolites that did not match entries in the internal database[4]. All metabolite identifications and quantification ions were validated and confirmed to reduce deconvolution errors during automated data-processing and to eliminate false identification. Peak areas for identified metabolites and unknowns were reported. The data were log2-transformed. There were 336 metabolites identified. Potential sample outliers were assessed using a robust Mahalanobis distance (RMD) measure based on correlation, median absolute deviation (MAD), and skewness and the proportion of missing values[6]. No potential sample outliers were identified. Normalization of the data was performed via global median centering. Analyses were performed in R version 4.2.0, utilizing the pmartR package[7].

**Reversed-phase and hydrophilic interaction liquid chromatography-tandem mass spectrometry**

Metabolite extracts were analyzed using liquid chromatography-electrospray ionization tandem mass spectrometry (LC-ESI-MS/MS) on a Waters H-Class UHPLC system coupled to a Thermo Q Exactive Plus Orbitrap, operating in both positive and negative ionization modes. For reverse-phase separation, compounds were resolved on a Thermo Hypersil GOLD column (2.1 × 150 mm, 3 μm particle size) at 30 °C and a flow rate of 400 μL min⁻¹. Mobile phase A (Water with 0.1% formic acid) and B (ACN with 0.1% formic acid) were initially 90:10, respectively. The gradient method continued as follows: 0–2 min held at 90% A; 2–11 min 10% A; 11–12 min 10% A; 12–13.5 min held at 10% A with an increased flow rate to 500 μL min–1; 13.5-14 min at 90% A flow rate at 500 μL min–1; 14-14.3 min at 90% A decrease flow rate to 400 μL min–1; 14.3-15 min held at 90% A. For hydrophilic interaction chromatography (HILIC), polar metabolites were separated using an ACQUITY UPLC BEH HILIC column (2.1 × 100 mm, 1.7 μm particle size) at 50 °C and a flow rate of 300 μL min⁻¹. Chromatography was adapted from Clendinen et. al 2019[8]. Mobile phase A (5% ACN: 95% 10 mM NH4OAc in H2O with 0.05% NH4OH) and B (100% ACN with 0.05% NH4OH) were initially 5% and 95%, respectively at a flow rate of 300 μL min–1. The gradient method continued as follows: 0–6.0 min. 63% A; 6.0-7.0 min 63% A; 7.0–7.1 min 5% A. 7.1-7.2 min. 5% A with an increased flow rate of 500 μL min–1; 7.2–9.5 min held at 5% A. 9.5-9.7 min. 5% A with a decreased flow rate of 300 μL min–1; and 9.7–10.0 min held at 5% A.

For MS analysis, the HESI source parameters included a spray voltage of 3.7 kV in positive mode and 3.0 kV in negative mode, with a capillary temperature of 320 °C. Full MS scans were performed at a resolving power of 140,000 FWHM at m/z 200, with a scanning range of m/z 80–800, AGC target of 3 × 10⁶ ions, and maximum injection time of 20 ms. Data-dependent acquisition (dd-MS²) included a resolving power of 17,500 FWHM at m/z 200, AGC target of 1 × 10⁵ ions, maximum injection time of 100 ms, isolation width of 0.4 m/z, loop count of 8, and normalized collision energies (NCE) of 20, 30, and 40 eV using higher-energy collision dissociation (HCD).

Thermo raw files were processed using Thermo Compound Discoverer 3.3, with metabolites identified via internal reference libraries and external databases such as MzCloud, GNPS, and MoNA. Spectra alignment used an adaptive curve with retention time tolerances of 0.3 min for RP mode and 0.6 min for HILIC mode, and a mass tolerance of 3 ppm. Peaks were selected based on a minimum intensity of 1.5 × 10⁴ and signal-to-noise ratio (S/N) of 3. Features were grouped by mass tolerance (3 ppm) and retention time tolerance (0.3 min) and excluded if not present in at least five samples compared to blanks. Compounds were annotated based on isotopic patterns, retention time, MS¹, and/or MS² data, followed by manual validation.

For each metabolite, any value below 1e5 was replaced with NA (missing). Subsequently, the data were log2-transformed. The identification of metabolites across different chromatography methods and ionization modes yielded the following results: 1930 metabolites were identified using reversed-phase chromatography in negative ion mode (RP_neg), and 2393 metabolites in positive ion mode (RP_pos). Using hydrophilic-interaction liquid chromatography, 4011 metabolites were identified in negative ion mode (HILIC_neg) and 2352 metabolites in positive ion mode (HILIC_pos). Normalization of the data was performed via global median centering. Potential sample outliers were assessed using a robust Mahalanobis distance (RMD) measure based on correlation, median absolute deviation (MAD), and skewness[6]. No potential sample outliers were identified. Analyses were performed in R version 4.2.0, utilizing the pmartR package[7].

To capture dynamic changes in metabolite abundances along the infection gradient across multiple acquisition modes in a reproducible and comparable manner, we automatically extracted Kyoto Encyclopedia of Genes and Genomes (KEGG) compound annotations from metabolites identified RP_neg, RP_pos, HILIC_neg, and HILIC_pos, and GC using a custom Python script. Normalized metabolite abundances were then linked to treatment group metadata, which defined an ordered progression of infection from group –7 (the lowest infection point) through group 3 (the highest infection point). For each compound, abundances were aggregated by treatment to calculate mean values across all fungal garden consortia replicates. To enable comparability across the five acquisition modes, these means were centered on the compound-specific dataset average and scaled to the maximum absolute deviation, producing normalized values between -1 and 1. This transformation emphasized the relative direction and magnitude of treatment responses while reducing the impact of mode-specific intensity differences. Compound-level heatmaps were generated for each metabolite (**Figure 2** and **Supplementary Figures 6 and 7**) and subsequently combined into multi-panel figures, allowing direct comparison of metabolite groups associated with KEGG pathways of interest. The metabolite integration and visualization workflow was implemented in R (version 4.5.1) as a custom function, with data wrangling carried out using the tidyverse suite (readr, dplyr, tidyr, stringr, purrr), and visualizations created with ggplot2 and patchwork.

**Multi-omics data integration**

Multi-omics data integration was manually performed by paring enzymes with metabolomic evidence to illuminate the conversion reaction of certain pathways, following the pathway reconstruction. As depicted in our **Figure 2** and **Supplementary Figure 7**, we used our multiomics data to reconstitute the parts of the following reference pathways: KEGG Degradation of aromatic compounds (map01220), KEGG Aminobenzoate degradation (map00627), KEGG Lysine degradation (map00310), KEGG Lysine biosynthesis (map00300), and KEGG Arginine biosynthesis (map00220).

**Supplementary Figure 1.** Representative image of a native fungal garden transferred into a tube after a portion of a healthy reference sample was collected. The fungal garden was subsequently infected with the pathogenic fungus *Escovopsis weberi* on one side of the tube, illustrating a spatiotemporal gradient of infection spreading across the garden. Representative images of a healthy fungal garden, characterized by the native *Leucoagaricus* fungus with its white, fuzzy appearance, are provided, along with the changes observed when *Escovopsis* is present, including a shiny, spider web-like texture. Example images of all six gardens are provided, showing the state of each garden on the day the infection was stopped, with clear visual comparisons between the healthy and infected sections.

**Supplementary Figure 2.** An example of the metaproteomic reference database that illustrates the comprehensiveness of our reference database with respect to taxonomic composition. Nodes are color-coded by taxonomic groups.

**Supplementary Figure 3.** Metaproteomic-informed functional characterization of native and pathogenic fungi across each tube, used to determine the interface zone for each garden. The interface was used to realign all gardens so that their treatment group 0 positions overlapped. Following alignment, 2 garden tubes (B_A and M_A) spanned from −7 to +3 relative to the interface (yielding 11 standardized treatment groups), with the remaining garden tubes having slightly fewer groups. Since the infection for garden M_B progressed further along the gradient due to the longer infection time (5days), samples M_B_9, M_B 10, and M_B_11 were removed to standardize the range to −7 to +3. Garden tubes where each garden colony is a unique biological replicate except the Mona Lisa colony, which was sampled twice: 1. April-A (A_A), 2. Bianca-A (B_A), 3. Joan-B (J_B), 4. Mona Lisa-A (M_A), 5. Mona Lisa-B (M_B), and 6. Donna-A (D_A).

**Supplementary Figure 4**. Metaproteomics-informed taxa distribution across the tubes, highlighting dynamic changes in taxa abundance based on protein and peptide profiles in response to infection-induced perturbation. A and B – The line plots represent the mean abundance of taxon-specific peptides and proteins, respectively, with error bars indicating the standard deviation of treatment group replicates from all six fungal garden consortia. C and D – The line plots represent the mean abundance of native fungus- and *Escovopsis*-specific peptides and proteins, respectively, with error bars indicating the standard deviation of treatment group replicates from all six fungal garden consortia.

**A	Substrate specificity: Lignin**

K20929, EC:1.2.3.15, Glyoxal/methylglyoxal oxidase

**B	Substrate specificity: Cellulose**

K05349, EC:3.2.1.21, β-glucosidase

K01225, EC:3.2.1.91, Cellulose 1,4-β-cellobiosidase

**C	Substrate specificity: Hemicellulose**

K01181, EC:3.2.1.8, Endo-1,4-β-xylanase

K01192, EC:3.2.1.25, Exo-β-D-mannanase

K07407, EC:3.2.1.22, α-galactosidase

K01209, EC:3.2.1.55, α-L-arabinofuranosidase

K19355, EC:3.2.1.78, Endo-1,4-β-mannanase

**D	Substrate specificity: Pectin**

K01051, EC: 3.1.1.1, Pectinesterase

K01728, EC:4.2.2.2, Pectate lyase

K18195, EC:4.2.2.23, Rhamnogalacturonan endolyase

K15530, EC: 3.1.1.86, Rhamnogalacturonan acetylesterase

K18106, EC:1.1.1.-, D-galacturonate reductase

**Supplementary Figure 5.** Bar graphs show the distribution of carbohydrate-active enzymes specific to A) lignin, B) hemicellulose, C) cellulose, and D) pectin, annotated to native fungi and/or *Escovopsis,* accompanied by heatmaps representing their corresponding peptide abundances across the tube. The bar graphs illustrate the taxonomic dynamic (purple-native fungus, gold-*Escovopsis*) of the specific enzyme across the infection gradient, calculated by the sum of the mean normalized abundance across all six garden consortia per treatment group, partitioned by taxon. Error bars indicate variation among replicate samples for each taxon-specific segment, shown as standard error and positioned relative to the corresponding stacked segment. The heatmap summarizes peptide abundance as the mean abundance across all six garden consortia for each treatment group.

**Supplementary Figure 6.** Heatmaps show the distribution and abundance of specific amino acids (arginine, lysine, glutamine, proline, histidine, tyrosine, phenylalanine) across the tubes, indicating the separation technique used for metabolite detection. The heatmap summarizes metabolite abundance as the mean abundance across all six garden consortia for each treatment group. To ensure comparability across different separation techniques, these means were scaled within compound-specific dataset, producing normalized values between -1 and 1.

**Supplementary Figure 7.** Multi-omics integration across all six fungal garden consortia unravels taxon-specific metabolic shifts within complex microbiomes over spatiotemporal scales. A – Reconstructed pathways for aromatic compound degradation. B – Reconstructed arginine metabolism pathway. C – Reconstructed lysine metabolism pathway. The bar graph illustrates the abundance change of the specific enzyme assigned to the two contrasting taxa, native fungus (purple) and *Escovopsis* (gold), across the infection tube. Peptides of each visualized enzyme that were uniquely detected in the respective taxon were shown in the heatmap next to the bar graph. The normalized and scaled metabolite abundances per separation technique were plotted across the treatment groups. The line plot displays the abundance shift of the key metabolite across sample replicates detected from a certain separation technique to highlight its consistent trend along the treatment group. Asterisks next to metabolite names indicate metabolite identification metrics[11] (i.e., 1 (high confidence) to 5 (low confidence)). Metabolites indicated with ***** correspond to level 1 confidence, whereas ***** represents level 2 confidence. Metabolites labeled with **^#^** indicate that one separation method was removed from the reported metabolite heatmap due to showing a distinct trend. This discrepancy may be attributed to interference from isobaric compounds. For 2,3-dihydroxybenzoate, the separation approach with confident MS/MS results was retained. For saccharopine, the separation approaches with well-resolved peak and confident MS/MS in ionization mode was retained.

References:

1.	Nicora CD, Sims AC, Bloodsworth KJ, Kim YM, Moore RJ, Kyle JE, et al. *Metabolite, Protein, and Lipid Extraction (MPLEx): A Method that Simultaneously Inactivates Middle East Respiratory Syndrome Coronavirus and Allows Analysis of Multiple Host Cell Components Following Infection.* Methods Mol Biol, 2020. **2099**: p. 173-194.

2.	Nakayasu ES, Nicora CD, Sims AC, Burnum-Johnson KE, Kim YM, Kyle JE, et al.
 *MPLEx: a Robust and Universal Protocol for Single-Sample Integrative Proteomic, Metabolomic, and Lipidomic Analyses.* Msystems, 2016. **1**(3).

3.	Burnum-Johnson KE, Kyle JE, Eisfeld AJ, Casey CP, Stratton KG, Gonzalez JF, et al.
 *MPLEx: a method for simultaneous pathogen inactivation and extraction of samples for multi-omics profiling.* Analyst, 2017. **142**(3): p. 442-448.

4.	Velickovic M, Wu R, Gao Y, Thairu MW, Velickovic D, Munoz N, et al. *Mapping microhabitats of lignocellulose decomposition by a microbial consortium.* Nature Chemical Biology, 2024. **20**(8).

5.	Suen G, Teiling C, Li L, Holt C, Abouheif E, Bornberg-Bauer E, et al. *The genome sequence of the leaf-cutter ant Atta cephalotes reveals insights into its obligate symbiotic lifestyle.* PLoS Genet, 2011. **7**(2): p. e1002007.

6.	Aylward FO, Burnum-Johnson KE, Tringe SG, Teiling C, Tremmel DM, Moeller JA, et al.., *Leucoagaricus gongylophorus produces diverse enzymes for the degradation of recalcitrant plant polymers in leaf-cutter ant fungus gardens.* Appl Environ Microbiol, 2013. **79**(12): p. 3770-8.

7.	Aylward FO, Burnum KE, Scott JJ, Suen G, Tringe SG, Adams SM, et al. *Metagenomic and metaproteomic insights into bacterial communities in leaf-cutter ant fungus gardens.* ISME J, 2012. **6**(9): p. 1688-701.

8.	Lee JY, Mitchell HD, Burnet MC, Wu R, Jenson SC, Merkley ED, et al. *Uncovering Hidden Members and Functions of the Soil Microbiome Using De Novo Metaproteomics.* J Proteome Res, 2022. **21**(8): p. 2023-2035.

9.	Nesvizhskii AI, Aebersold R. *Interpretation of shotgun proteomic data: the protein inference problem.* Mol Cell Proteomics, 2005. **4**(10): p. 1419-40.

10.	Van Den Bossche T, Kunath BJ, Schallert K, Schape SS, Abraham PE, Armengaud J, et al.
 *Critical Assessment of MetaProteome Investigation (CAMPI): a multi-laboratory comparison of established workflows.* Nat Commun, 2021. **12**(1): p. 7305.

11.	Schymanski EL, Jeon J, Gulde R, Fenner K, Ruff M, Singer HP, et al.
 *Identifying Small Molecules via High Resolution Mass Spectrometry: Communicating Confidence.* Environmental Science & Technology, 2014. **48**(4): p. 2097-2098.
